# Comments on two recent publications on GM maize and Roundup

**DOI:** 10.1038/s41598-018-30440-7

**Published:** 2018-09-03

**Authors:** Dennis Eriksson, Klaus Ammann, Bruce Chassy, Aakash Chawade

**Affiliations:** 1 Department of Plant Breeding, Swedish University of Agricultural Sciences, Alnarp, Sweden; 2 Institute of Plant Sciences, University of Bern, Bern, Switzerland; 3 Department of Food Science and Human Nutrition, University of Illinois, Urbana, USA

## Abstract

Two -omics studies on genetically modified maize and Roundup-fed rats, recently published in the journal Scientific Reports, contain serious flaws in the experimental design, methodology and interpretation of results, which we point out here. The use of -omics technologies are of increasing importance in research, however we argue for a cautious approach to the potential application in food safety assessments as these exceptionally sensitive and complex methods require a thorough and detailed evaluation of the biological significance of obtained results. Arising from: Mesnage *et al*. Sci Rep 7:39328 (2017), Mesnage *et al*. Sci Rep 6:37855 (2016).

## Introduction

Two studies recently published in Scientific Reports^1,2^ contain a number of questionable issues related to the experimental design and the interpretation of the obtained data. We here point these out and discuss why caution about the far-reaching conclusions presented in the two studies is necessary. The first study (here called the “GM maize report”) claims that genetic transformation process caused metabolic disturbances in genetically modified (GM) NK603 Roundup-tolerant maize, questioning the compositional similarity of this cultivar to a non-GM control cultivar^1^. The second study (here called the “Roundup report”) reports that exposure of rats to an ultra-low dose of the glyphosate-based herbicide Roundup were found to cause significant metabolome and proteome changes allegedly indicative of liver dysfunction^2^. Both reports are based on samples collected from an earlier two-year study^3,4^.

The GM maize report does not provide evidence in support of the claim that the transformation process has caused the observed metabolic differences. First, no evidence is presented to prove that DKC2678 (Roundup-tolerant NK603) is near-isogenic to DKC 2675. Similar identity numbers in a seed catalogue (Materials and Method, p. 10^1^) is no guarantee that the two cultivars are genetically near-identical. The appropriate control comparator should have been a known near-isogenic, or, preferably, a progenitor line. Lack of standardised protocols such as randomised block field design precludes the evaluation of possible confounding environmental factors, which are often found to have a greater impact on variation than transgenesis^5,6^. The lack of spatially separated biological replicates does not conform to standard experimental design, and the use of only two temporal replicates undermines statistical power. It is not clearly described how the technical replicates were obtained, neither is the merging of the two biological replicates for the metabolome analysis explained (Figure 1, p. 3; Materials and Method, p. 10–11^1^). The authors cite one study (Frank *et al*.^5^) which reported that approximately 3% of metabolites in NK603 maize kernels differed compared to isogenic lines, however without mentioning that the cited study itself explained that natural variability and environmental factors were the dominant parameters driving the variability of the maize metabolite profiles. The authors do acknowledge that further experiments made under different environmental conditions are needed (Discussion, p. 8^1^) but nevertheless proceed with over-sensationalist claims that the transformation process caused a general disturbance and a metabolic imbalance in energy and carbohydrate metabolism in the GM plants (Title; Discussion, p. 10^1^). There is also no reference to the expected natural variation in the levels of selected proteins and metabolites among maize cultivars, severely hampering any conclusion about the observed differences. The polyamines cadaverine and putrescine are highlighted in the GM maize report as being of potential negative health concern, however maize kernels normally contain a wide range of these substances, which may even be beneficial at moderate levels^7^. In our opinion, the study lacks a suitable experimental design to prevent the influence of the environment, and lacks references to the natural variation of these compounds in maize. Thus, the study does not allow the authors to argue, as they do (e.g. Discussion, p. 10^1^), that the nutritional quality has been hampered by metabolic imbalances nor that the observed differences are due to the transformation process and the resulting expression of a transgenic protein. In conclusion, it is not possible from this study to determine the cause of the observed differences between the two maize varieties, or to link these results to any effect of biological relevance.

In the Roundup report, it is relevant to note that the analysed liver samples came from the same rat material used for a several years old study^3^ which has been criticised for the use of Sprague-Dawley rats which display a high rate of age-related spontaneous tumours, flawed statistical analysis and low statistical power due to the small sample size, and lack of experimental details to scientifically assess the quality of the study^8–10^. In the statistical analysis of the metabolome data, the authors chose to reduce the type II error (false negatives) by looking at the 55 metabolites that were significant at the more lenient p-value threshold (p < 0.05) instead of correcting for multiple comparisons as in the proteome analysis, while the Benjamini-Hochberg False Discovery Rate (FDR) analysis only yielded three significantly different metabolites at a threshold of q < 0.05 (Results, p. 6; Discussion, p. 9^2^). The p-value threshold (p < 0.05) implies that 34 (5%) of the 673 analysed compounds could be obtained by chance alone in two identical situations and thus could be false positives, making the conclusions unreliable without further testing and validation. Though not completely incorrect, we would emphasise that this approach calls for careful treatment and interpretation of the results. In conclusion, the choice of sample material for the experiments, the statistical analysis and the interpretations are questionable. The authors do acknowledge that small sample size and aged animals give little statistical power (Discussion, p. 9^2^), but nonetheless go ahead with speculative conclusions (Title; Abstract; Discussion, p. 7; Conclusions, p. 10^2^).

Both reports claim disproportionate biological implications based on the presented -omics data. We recommend the authors to acknowledge the wealth of studies with contradictory results. Of relevance to -omics research related to GM crops, an article was cited in the GM maize report that evaluated 44 publications containing -omic profiling studies, of which twelve were on GM maize. It concluded that natural variability, conventional breeding techniques and environmental factors consistently have a higher impact on gene expression, protein and metabolite levels than transgenesis^11^. The evaluation was later updated to include a total of 60 GM crop profiling -omic studies that reinforced the original conclusion^12^. More recent studies^13,14^, along with a consensus in the scientific community^15^, also confirm this general perspective. The authors seem to misinterpret the Substantial Equivalence concept, which recognizes that differences between varieties will always be observed, providing a starting-point of the food safety evaluation rather than an end-point in itself. Any observed differences indicated by molecular profiling are not necessarily biologically meaningful in a health and safety context, and rather than using a single comparator it would be more appropriate to develop a reference class model covering variety, growth location, replications and other sources of variation^16^.

Both GM crops and glyphosate are currently subjects of political discussion in the European Union. Since the accumulated scientific body of evidence can (and should) be used to guide policy decisions, it is imperative that the research is solid and indisputable. While we support the research and development of cost-effective and accurate methods for safety assessment of food and feed crops, we disagree with the claims of the GM maize and Roundup reports since the problems with the experimental design and data interpretation prevent the experimental evidence to support the conclusions. Ultimately, the wider discussion concerns the standards of scientific publication. Researchers are constantly balancing between the urge to publish generated data and the requirements to further validate, or reject, their hypotheses through repetition and potential generalisation. One important guideline is that the more spectacular a finding is, the more rigorous are the requirements for validation. It is against this background that we wish to question the claims of the two reports criticised here and weigh their presented findings against the accumulated body of evidence in the research area.
